# Recognizing the Less Explored “Active Solid”–“Moving Liquid” Interfaces in Bio/Chemical Sensors

**DOI:** 10.1021/acssensors.3c00773

**Published:** 2023-07-14

**Authors:** Nikhil Bhalla

**Affiliations:** † Nanotechnology and Integrated Bioengineering Centre (NIBEC), School of Engineering, Ulster University, 2-24 York Street, Belfast, Northern Ireland BT15 1AP, United Kingdom; ‡ Healthcare Technology Hub, Ulster University, 2-24 York Street, Belfast, Northern Ireland BT15 1AP, United Kingdom

**Keywords:** solid−liquid interfaces, biosensors, transducers, continuous flow, plasmonics, electrochemistry

## Abstract

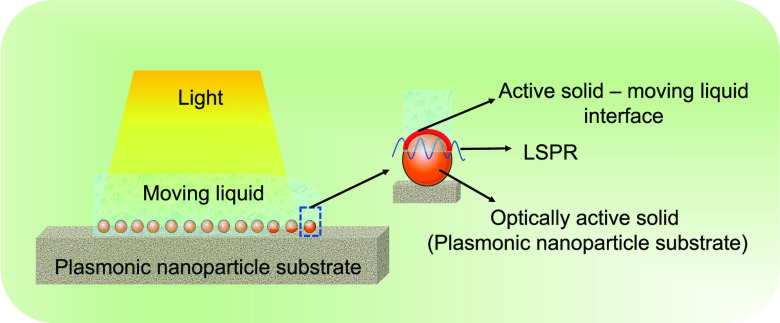

Bio/chemical sensors possess a plethora of advantageous features that have proven to be invaluable tools in detecting and monitoring biomolecules, facilitating advancements in healthcare, environmental monitoring, food safety, and more. However, when it comes to their routine use in continuous fluid flow conditions, an intricate web of solid–liquid interfacial phenomena emerges, which requires a deep understanding of the sensor surface and fluid interations. These interfacial phenomena encompass a broad spectrum of physical, chemical, and biological processes, other than the actual detection, that influence the sensor’s response. In this context, perhaps exploring a new theme “active solid”–“moving liquid” interface will unleash the full potential of bio/chemical sensors in any flow-based application.

Performing bio/chemical sensing in continuous fluid flow conditions can alter the dynamics of a chemical reaction under investigation, leading to variations in reaction rates or binding activity at different fluid flow rates. On the other hand, the transducer element of a bio/chemical sensor is intrinsically sensitive to external stimulus applied on the sensor surface. This means that any bio/chemical sensor will reveal changes in its output signal when disturbed by liquid, in static or continuous flow condition, that is in contact with the sensor surface. Therefore, it is important to isolate the physical effects, such as friction from the liquid, liquid-slips, contact electrification, or local temperature changes that could be occurring on the solid surface of the sensor, other than the actual bio/chemical detection event. Moreover, such physical effects are immensely augmented in the dynamic state of the sensor, i.e., when the fluid is continuously moving on the surface, and therefore such a system requires a much deeper understanding of effects associated with moving fluid/flow rates on the sensor response.

To isolate such effects and especially in continuous fluid flow conditions, it is absolutely essential to investigate the solid–liquid interfacial phenomenon arising due to the interaction of the sensor surface with the liquid in contact with it. However, a couple of questions which then arise are (1) how can one isolate the effects of this solid–liquid interaction on the surfaces of bio/chemical sensors and (2) are there mathematical or physical models in literature that one should incorporate in the equations of sensors which will exclusively relate output of the sensor to the bio/chemical detection activity? To address both of these questions, the development of new models that quantify the interaction between the sensor surface and the moving liquid is imperative. Once these new models are established, the equations describing the transduction mechanisms of bio/chemical sensors must be modified (to incorporate the effect of moving fluid) for yielding true detection signals for the bio/chemical event happening under the continuous fluid flow conditions.

Traditionally, several routinely used continuous fluid flow based bio/chemical sensors such as quartz crystal microbalance (QCM) and surface plasmon resonance (SPR) have neglected the effect of continuous moving fluid on their surfaces. As a result, these sensors have reported different results for parameters which indicate binding kinetics.^1−3^ This is because wavelength/frequency changes within such sensors are interpreted exclusively with mass change on the sensor surface, irrespective of the flow conditions. The repercussion of this slender cognizance of solid–liquid interfacial phenomenon is manifested in the form of overestimation/underestimation of mass which binds on the surface of the sensor. There are several examples of various transducers which are reported to overestimate the amount of analyte detected when correlated with the equations/models that govern their signal response.^4,5^ Nevertheless, recent works have discovered events such as “liquid slip” previously totally ignored in the response of QCM sensor.^6,7^ Along similar lines, it remains to be understood if such effects exist in a range of electrochemical and optical sensors that measure binding kinetics in continuous flow conditions of the fluid.

## Impact of Ignoring the Moving Fluid and Sensor Surface Interaction

There is a profound impact of neglecting the effect of continuous moving fluid in sensors, and it may urge researchers to reassess the analyte’s “fraction bound” on the sensor surface when it is considered proportional to the sensor signal.^8^ This is because most of the analyte fraction bound is presented in the form of *K*_d_/*K*_on_/*K*_off_ which are measured in continuous flow conditions. Here, *K*_on_ is a constant used to indicate how fast the analyte binds to its target, while *K*_off_ is a constant used to specify how fast the analyte dissociates from its target. The *K*_d_ is simply a ratio of *K*_on_:*K*_off_ which typically quantifies the affinity or strength of binding of a single analyte to its target.^9^

It is well-known that these reaction rate constants are dependent on the conditions such as the buffer pH, temperature of bioentities and the buffer, and the composition of the buffer itself.^9,10^ For instance, experiments conducted with Biacore SPR system at different temperatures (ranging from 20 °C to 37 °C) yield different binding rates after the SPR sensorgrams are analyzed.^10^ The conundrum of such a sensor outcome is to precisely isolate the effects of temperature on the transducer response from the thermodynamic effect of the binding interaction. In parallel, if the transducer is sensitive to some of the aforementioned conditions such as the pH and buffer content (type of ions in the buffer), the response of the sensor becomes more complicated to reveal the exclusive contribution of the binding event. As mentioned earlier, such effects are amplified in the dynamic state of the sensor, i.e., when fluid is moving, which requires a much deeper understanding of fluid flow rate effects on the sensor response.

Significant backlash of limited understanding of such solid–liquid interfacial phenomenon on sensor surfaces has also led to neglection of fluid flow rates in determination of binding constants within the academic community. For example, within the experiments conducted in the work of MacDonald et al.^3^ a flow rate of 30 μL·min^–1^ for SPR measurements and a flow rate of 50 μL·min^–1^ for QCM measurements was used to elucidate binding events. Thereafter, both SPR and QCM results were directly compared with each other in the context of binding events. In this case, unless the flow rates were kept the same, signals from SPR and QCM cannot be directly compared without caution. Additionally, some works in the past have also claimed enhanced sensor sensitivity by simply observing larger sensor signal output at specific flow rates without considering the exclusive effect of flow on the sensor surface.^11^

Another major impact of not considering the physical effects of continuous flow rate is that different industries selling the same chemical (especially in the case of proteins, enzymes) may report different *K*_d_/*K*_on_/*K*_off_ values in their data sheets without providing much information on the fluid flow rates and the type of instrument used for computing the binding constants. Typically, a molecule may not have different binding constants when evaluated with same instruments unless tested in different flow rates. This may result in academic researchers or other clients of the company buying reagents which show higher binding affinity. This might be beneficial to a company from a commercial perspective, but researchers buying the reagents may want to investigate how binding constants reported in the data sheets were actually calculated—distinctly understating what flow rates and which type of instrument/transduction mechanism were used to report the binding constants in the data sheet. This will allow researchers to avoid ending up with unexpected results, especially when there are strict timelines for delivery of a project from the funding body.

## Is Sensor Calibration/Dose Response Enough to Mitigate the Impact of Solid–Liquid Interface?

The argument of the bio/chemical sensing community to address the fundamental issues associated with liquid (in static and dynamic state) on top of the sensor surface is that the physical effects at the solid–liquid interface are manifested linearly for a given flow condition. Therefore, if a sensor is developed to detect a certain biomolecule of interest, the process of calibration of a sensor in the form of dose response (if the flow rate, fluid temperature, and other conditions are unchanged by the user) will nullify the contribution from the physical effects arising locally at the interface of solid and liquid. This is because the individual sensor response at different analyte concentrations would consist of the same error generated in a particular fluid flow condition.

There is absolutely no problem with interpretation of sensor results using calibration, but this limits use of a sensor in specific conditions, the conditions for which the sensor was developed, and a sensor calibration becomes mandatory prior to its use in a different flow condition. A potential solution to this is to investigate the effects of flow on a plethora of electrochemical and optical transducers that are used in continuous fluid flow conditions, including the microfluidics technology for bio/chemical sensing.

## “Active Solid”–“Moving Liquid” Interface in Bio/Chemical Sensors

**Figure 1:**
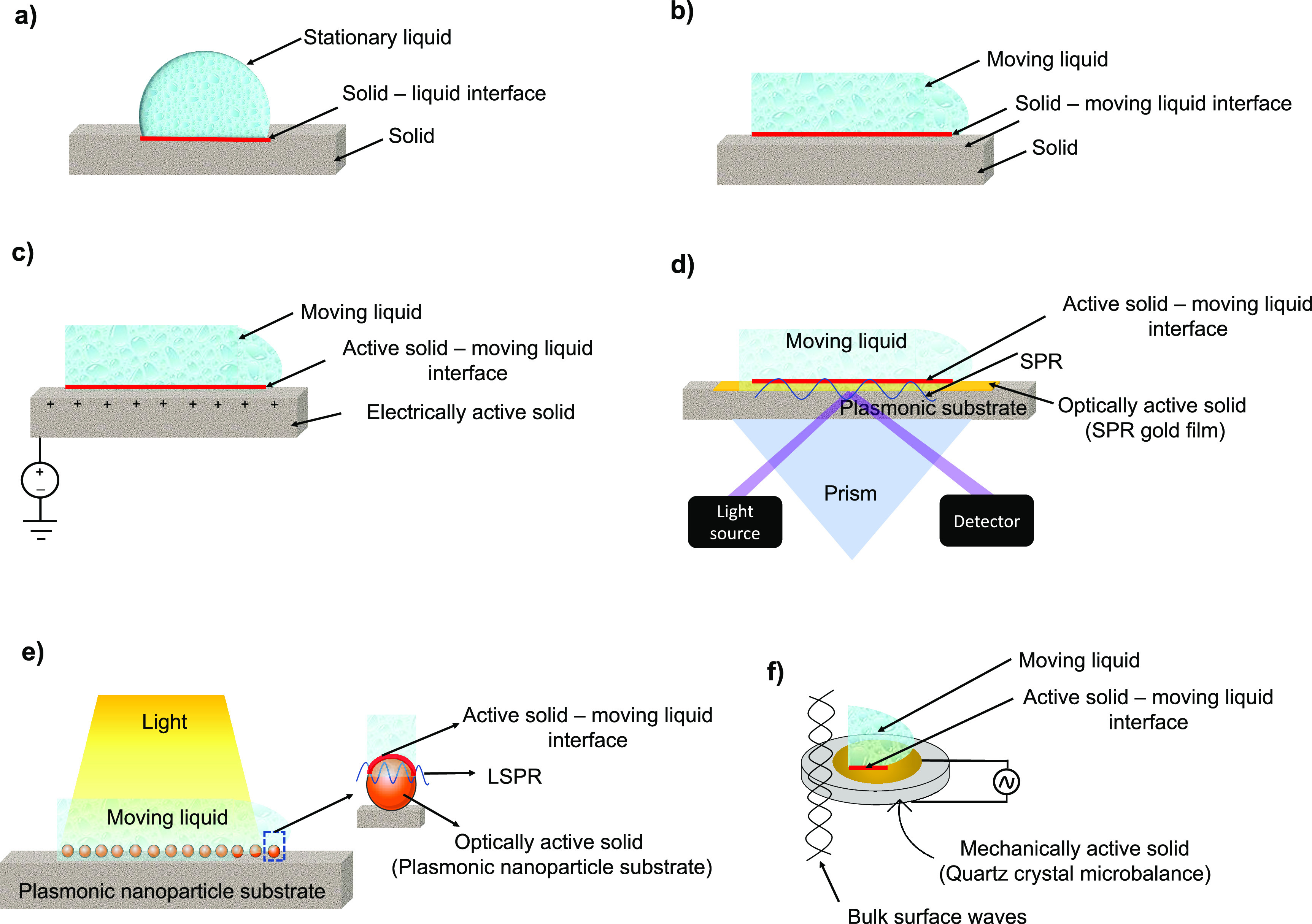
Solid liquid interfaces in bio/chemical sensors: (a) shows a solid–liquid interface between a stationary liquid resting on the solid surface and the solid surface itself. (b) demonstrates a concept of solid–moving liquid interface between the liquid moving on the solid surface and the solid surface. (c) demonstrates an active solid–moving liquid interface. Here the solid is electrically active, and the liquid is in a moving state. Such interfaces typically exist in a three-electrode biosystem comprising counter electrode, working electrode, and reference electrode used in continuous flow conditions. (d) is interface of a surface plasmon resonance (SPR) chip comprising gold nanofilm, considered as optically active solid, with the moving liquid. (e) shows an interface of a localized surface plasmon resonance (LSPR) chip comprising a nanostructured surface, considered as an optically active solid, with the moving liquid. (f) depicts a quartz crystal microbalance (QCM) which mechanically vibrates when alternating current is applied to it, and henceforth, it can be considered a mechanically active solid or a simply a vibrating solid, presenting a mechanically active solid–moving liquid interface.

In the context of the aforementioned bio/chemical sensing in continuous fluid flow conditions, a value proposition for introduction of a new term for sensor surfaces, i.e., “active solids” instead of simply solids, is created; for details, see Figure 1 and Table 1. This is because a sensor surface is in an active state compared to a simple solid without any external sensor. See Figure 1a and b, demonstrating a simple solid (inactive state) in contact with liquid in static and moving state, respectively. In contrast, Figure 1c shows an electrically active solid, Figure 1d,e shows optically vibrating SPR/LSPR solid, respectively, and a mechanically vibrating active solid in the form of QCM is shown in Figure 1f. Note that in Figure 1c–f the interface between the active solid and the moving liquid is also shown. The moving liquid requires a completely new recognition of solid–liquid interracial interactions in the fluid dynamics community where solids are usually modeled in a recessive state, unlike solids within transducers which are in active state (such us in a vibrating state). Henceforth, scrutinizing these effects at “active solid”–“moving liquid” interface should yield development of new models/equations which will precisely govern how bio/chemical sensors operate in flow. This may as well lead to a new research field of “active solid–moving liquid interface”. Some physical effects occurring at the interface of the “active solid”–“moving liquid” interface are shared in Table 2.

**Table 1:** Definitions of Terms in the Context of Bio/Chemical Sensors and Solid_-Liquid Interface

	Term	Definition
1.	Solid	State of matter that has a definite shape and volume of its own.
2.	Active solid	Solid, to which a controlled external energy is applied (such as voltage or light) to make it electrically or optically active. Note that forms of energy other than electrical or optical can also be applied to make solids active.
3.	Liquid	Liquid is a state of mater which has a definite volume, but it takes the shape of the container.
4.	Moving liquid	Liquid, which is continuously flowing at a specific flow rate, unlike the stationary liquid which does not move.
5.	Solid–liquid interface	It is a boundary region separating a solid from a liquid which are in contact with each other. Its extension is strongly dependent on the properties of the interphase. For poorly soluble solids and small-molecule liquids such as water, it can spread over a thickness of the order of a few nanometers.
6.	Active solid–moving liquid interface	It is a boundary region separating an active solid from a moving liquid which are in contact with each other.
7.	Transducer	The transducer is an element that converts the biorecognition event into a measurable form of signal.
8.	Sensor surface	Solid top surface of the bio/chemical sensor which is in contact with liquid/moving liquid consisting of the target analyte.

**Table 2:** Definitions of Solid–Liquid Interfacial Processes

	Term	Definition
1.	Friction	Damping force or a dissipative force due to interplay of viscosity of the liquid and the roughness of the solid surface.
2.	Shear force	Force acting on the moving fluid parallel to the solid surface.
3.	Liquid slip/slip length	The slip length, more often used to describe the liquid slip is the distance beyond the solid–liquid interface where the liquid velocity linearly extrapolates to zero.
4.	Contact electrification	A phenomenon whereby solid surfaces become electrically charged upon contact with the liquid.
5.	Fluid inertia	The solid like behavior of the moving liquid’s first few layers when in contact with the solid/active solid.
6.	Surface tension	The virtue of liquid at rest to shrink into the minimum surface area possible when in contact with the solid surface.

**Figure 2:**
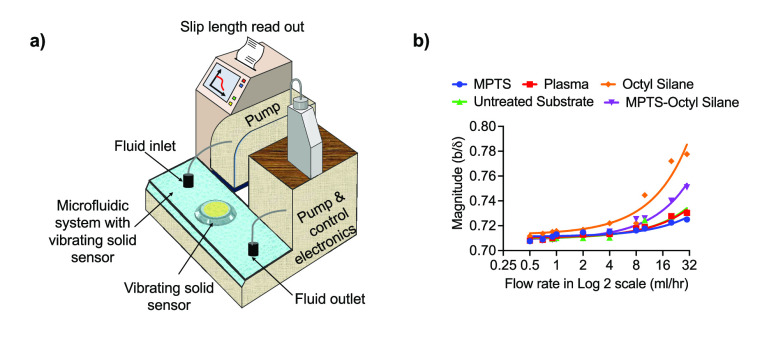
Liquid slip in active solid–moving liquid interface: (a) shows an experimental setup consisting of vibrating solid, microfluidic system, readout, and control electronics for studying effect of continuous fluid flow on the quartz crystal microbalance. (b) shows how the magnitude of liquid slip length changes with change in the fluid flow rates when the surface of QCM is treated with MPTS-octyl silane, octyl silane, MPTS, and plasma. It also includes the untreated QCM surface. Both subfigures are reprinted with permission from ref (6). Copyright (2022) Springer Nature.

A recent example of such a study is demonstrated by Payam et al.^6^ Here, the authors discover a “missing liquid slip” in the vibrating solid–liquid systems such as the QCM-based biosensor (Figure 2a shows their system) where traditionally frequency and dissipation changes were interpreted exclusively with mass change on the sensor surface, irrespective of the flow conditions. To some extent the study also argued the origin of the liquid slip by correlating the slip to a large extent with the flow rate of the liquid, rather than the surface wetting conditions. This was in contrast with the understanding of the liquid slip, where it is exclusively associated with the degree of surface wetting. For instance, a hydrophobic surface is well-known to provide a higher slip than a hydrophilic surface. While the experiments and theory within their work agreed to this concept, the work clearly demonstrated that the origin of slip is attributed more to the movement of the liquid. It can be concluded that with a change in the flow rate of the liquid, the slip changes to a much larger extent. The wetting condition then is simply a slip modulation factor. The second important finding was that the properties of the solid did not fully determine the slip of the Newtonian liquids. This means that hydrophilic surfaces are not slip-free. Slip values (in magnitude) comparable to hydrophobic surfaces can exist on active/vibrating solid surfaces at flow rates which are well within the laminar regime, as shown in Figure 2b. This finding is important for interpreting bio/chemical sensing results in flow. Additionally, the effects of liquid slip on energy dissipation and inertia were elucidated. A nonlinear solid-like behavior of the liquid was demonstrated in terms of inertial length. Essentially, the work has put forward the case that the first few layers of the liquid on a solid have mechanical characteristics like a solid, and this so-called “inertial length” increases as the flow rate increases (meaning more liquid layers are solid-like at higher flow rates). Such effects would completely change the perspective of how bio- and chemical sensor responses are interpreted in flow. Overall, the findings presented a new mathematical expression for liquid slip in the form of a complex number. The real part of the expression demonstrates the effect of flow (modulation in liquid properties), and the condition of the active solid surface (modulation of solid properties) is reflected by the imaginary part of the complex number.

## Concluding Remarks

Going forward, more of such works are required to understand active solid-moving liquid interactions. This will eventually revolutionize the current biosensing measurements, where most often the sensor response is associated with a single stimulus. Furthermore, it will also provide more informed evidence of binding kinetics of biomolecules in flow instead of directly associating the sensor response (including the sensitivity of the sensor) with binding events, irrespective of the flow conditions. The holistic impact of such studies on the bio/chemical sensing community will be reflected in presenting a much stronger case for the adoption of bio/chemical sensors as reliable and routine tools in widespread applications ranging from healthcare, environmental, and energy domains.
